# Space radiation exposure persistently increased leptin and IGF1 in serum and activated leptin-IGF1 signaling axis in mouse intestine

**DOI:** 10.1038/srep31853

**Published:** 2016-08-25

**Authors:** Shubhankar Suman, Santosh Kumar, Albert J. Fornace, Kamal Datta

**Affiliations:** 1 Department of Biochemistry and Molecular & Cellular Biology and Lombardi Comprehensive Cancer Center, Georgetown University, Washington, DC 20057, USA; 2 Center of Excellence in Genomic Medicine Research (CEGMR), King Abdulaziz University, Jeddah, Saudi Arabia

## Abstract

Travel into outer space is fraught with risk of exposure to energetic heavy ion radiation such as ^56^Fe ions, which due to its high linear energy transfer (high-LET) characteristics deposits higher energy per unit volume of tissue traversed and thus more damaging to cells relative to low-LET radiation such as γ rays. However, estimates of human health risk from energetic heavy ion exposure are hampered due to lack of tissue specific *in vivo* molecular data. We investigated long-term effects of ^56^Fe radiation on adipokines and insulin-like growth factor 1 (IGF1) signaling axis in mouse intestine and colon. Six- to eight-week-old C57BL/6J mice were exposed to 1.6 Gy of ^56^Fe ions. Serum and tissues were collected up to twelve months post-irradiation. Serum was analyzed for leptin, adiponectin, IGF1, and IGF binding protein 3. Receptor expressions and downstream signaling pathway alterations were studied in tissues. Irradiation increased leptin and IGF1 levels in serum, and IGF1R and leptin receptor expression in tissues. When considered along with upregulated Jak2/Stat3 pathways and cell proliferation, our data supports the notion that space radiation exposure is a risk to endocrine alterations with implications for chronic pathophysiologic changes in gastrointestinal tract.

Qualitatively, space radiation includes mostly high-energy particle radiation consisting mostly of energetic protons and heavy ions^1^. In contrast, terrestrial radiation except for radon gas is mostly photon radiation such as γ- and x-rays, which are sparsely ionizing radiation and less damaging than space radiation and are known in radiobiological term as low linear energy transfer (low-LET) radiation^2^,^3^,^4^,^5^. In space, energetic heavy ions such as ^12^C, ^16^O, ^28^Si, and ^56^Fe due to their high mass, charge, and energy cause dense ionization events along their primary traversal-track. Such radiation also generates secondary ionization (delta rays) events in tissues and radiobiologically heavy ion radiation is known as high linear energy transfer or high-LET radiation^6^. Importantly, energetic heavy ions are major contributors to the dose equivalent in galactic cosmic radiation (GCR), which originates from outside solar system and is continuously present in space^7^. Exposure to energetic heavy ion radiation due to its highly damaging high-LET characteristics remains a major health concern for astronauts undertaking long duration space missions beyond low earth orbit (LEO) such as a mission to Mars^7^. Importantly, unlike other radiation types in outer space, current shielding is unable to provide effective protection against energetic heavy ions raising further concerns about astronauts’ health during and after prolonged space travel^8^. With increasing interest in space tourism, energetic heavy ion radiation exposure is not only of concerns for astronauts but also for aspiring future space travelers at large^9^. The current study is focused on gastrointestinal (GI) tract because globally colorectal cancer (CRC) is the third most common cancer and in the USA, it is the second leading cause of cancer deaths. Therefore, even a modest increase above the already high spontaneous incidence of CRC after energetic heavy ion radiation exposure will have significant ramification not only for astronauts’ health risk estimates but also for future NASA deep-space exploration planning. Study into the effects of heavy ions on GI is also important due to the fact that GI homeostasis is critical for astronauts’ nutrition during prolonged space missions. However, literature on short- and long-term tissue-specific effects of heavy ion radiation including ^56^Fe radiation are still limited and a search of the PubMed using search term ‘56Fe radiation’ generated a list of only 148 publications. Dearth of biological data especially *in vivo* human or animal data has created a large gap in our understanding of energetic heavy ion radiation exposure-associated risk of GI pathologies including CRC. Therefore, our project is focused on delineating altered spectrum of post-heavy ion irradiation biological endpoints known to be associated with altered GI homeostasis and CRC. There are limitations in deriving statistically meaningful risk estimates using human data due to small number of astronauts. The goal is to acquire *in vivo* biological data in animal models for development of risk models, which NASA will be able to use for prediction of risk to human during long duration space missions. While more studies are required to develop a clear understanding of tissue-specific effects of ^56^Fe radiation, we have published a series of peer-reviewed original research papers on heavy ion exposure-associated GI tissue effects using CRC mouse model as well as wild type mice^3^,^4^,^5^,^10^,^11^,^12^,^13^,^14^,^15^. In the current study, we investigated leptin and IGF1 signaling due to our observation of increased obesity-related factors such as body weight, serum triglyceride and insulin, and peroxisome proliferator-activated receptor γ (PPARγ) after ^56^Fe radiation. Notably, PPARγ is a key regulator of adipocyte differentiation and it has been implicated in chronic diseases including obesity, diabetes, and cancer^16^,^17^. Additionally, increased body weight as well as increased leptin and IGF1 signaling are positively correlated with GI pathologies including inflammatory bowel disease and cancer.

Leptin encoded by obese gene (Ob) has been shown to regulate energy homeostasis, food intake, and body weight. Leptin upon binding to its receptor (ObR), activates receptor associated JAK2 and activated JAK2 in turn phosphorylates ObR at multiple sites allowing activation of downstream proliferative pathways such as STAT3, and STAT5^18^. In contrast, adiponectin via its receptors adiponectin receptor 1 (AdipoR1) and 2 (AdipoR2) opposes the effects of leptin on cells and has been demonstrated to be anti-proliferative and pro-apoptotic^18^,^19^. Adiponectin has also been reported to crosstalk and antagonize leptin pathway via activation of phosphatases PP2A and PTP1B leading to inhibition of proliferative Akt and JAK2 pathways respectively^18^,^19^. Additionally, the leptin pathway through JAK2 and insulin receptor substrates (IRS) has been demonstrated to crosstalk with the insulin-like growth factor 1 (IGF1), which along with its receptor IGF1R is expressed in small intestine as well as in colon. Both activated JAK2 and ligand-bound IGF1R activates IRS, which subsequently was shown to activate the PI3K/Akt pathway^20^. There is increasing evidence that leptin/IGF1 signaling cooperates to promote cellular proliferation and carcinogenesis, and altered metabolic/hormonal state and increased body weight^20^,^21^,^22^,^23^,^24^,^25^,^26^.

Both radiation exposure and metabolic/hormonal dysregulation have been linked to altered intestinal homeostasis and colorectal carcinogenesis^25^,^27^,^28^,^29^,^30^,^31^,^32^,^33^,^34^. Epidemiological observations linking radiation and colorectal cancer (CRC) are also well supported by studies in animal models^4^,^35^,^36^. Apart from carcinogenesis, radiation exposure has also been reported to cause metabolic and hormonal alterations related to growth and proliferation in human as well as in animal studies^37^,^38^,^39^,^40^,^41^,^42^,^43^,^44^,^45^. However, studies linking energetic heavy ion space radiation, CRC, and altered hormonal/metabolic state are limited. Here we demonstrate for the first time that exposure to a non-lethal dose (1.6 Gy) of energetic ^56^Fe ions led to increased serum IGF1, IGF binding protein 3 (IGFBP3), and leptin levels at two and twelve months after radiation exposure. We also show that radiation exposure was associated with increased IGF1R and Ob-R, and decreased AdipoR1 and R2 expression in intestine and colon tissues. When considered along with upregulated signaling pathways downstream of IGF1 and leptin, and increased proliferative markers and body weight in irradiated mice, our results are suggestive of increased risk for metabolic alterations and cancer in GI tract after energetic heavy ion radiation exposure.

## Results

### Increased obesity-related factors were observed 12-month after ^56^Fe radiation exposure

**Figure 1:**
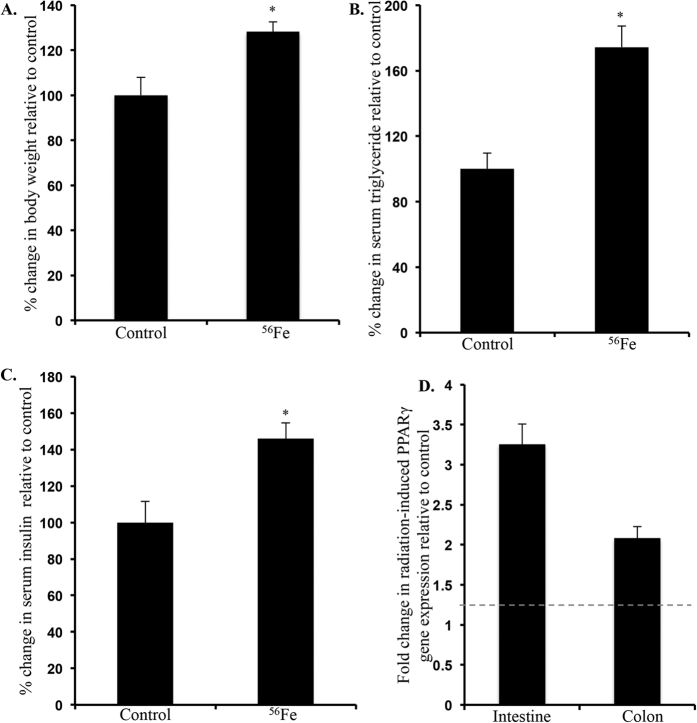
Increased body weight (**A**), serum triglyceride (**B**) and insulin (**C**), and intestine and colon tissue expression of PPARγ gene (**D**) was observed after ^56^Fe radiation. For PPARγ expression (**D**), the fold change (dotted line) and p-value cutoff for irradiated group was 1.25 and p < 0.05 respectively relative to control.

Obesity has been linked to chronic disease processes including CRC. Increased body weight (Fig. 1A) observed in ^56^Fe-irradiated mice led us to assess additional obesity-related factors. Exposure to ^56^Fe radiation was associated with increased serum triglyceride (TG; Fig. 1B) and insulin (Fig. 1C) levels. Additionally, when compared with γ radiation results, we observed that heavy ion radiation exposure was associated with higher body weight, and greater serum TG and insulin levels (Figure S1). We also observed increased expression of PPARγ in intestine and colon tissue 12-month after ^56^Fe radiation exposure (Fig. 1D).

### Heavy ion radiation exposure increased free IGF1 level in serum and IGF1R level in tissues

**Figure 2:**
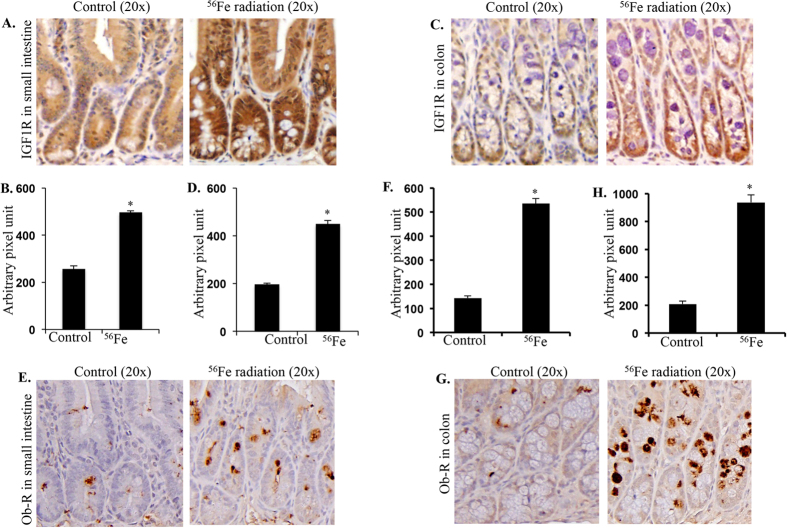
Altered receptor expression twelve months after energetic ^56^Fe exposure. Increased expression of IGF1R was observed in intestine (**A,B**) and colon (**C,D**) twelve months after radiation exposure. We also observed increased leptin receptor (Ob-R) expression was observed in intestine (**E,F**) and colon (**G,H**) after heavy ion radiation exposure.

Heavy ion ^56^Fe radiation led to increased serum IGF1 levels two months after exposure (Table 1). On the contrary serum IGFBP3 levels, although showed increasing trend after exposure, was not significantly higher relative to control (Table 1). The IGF1/IGFBP3 molar ratio indicating free IGF1 was slightly but significantly greater relative to control (Table 1). Although there are six IGFBPs, literature suggests that mainly IGFBP3 is involved in controlling bioavailability, activity, and distribution of free IGF1 through high-affinity IGF1/IGFBP3 complex formation^25^,^46^. We have measured total IGF1 (both bound and free) and IGFBP3 (both bound and free) by ELISA and calculated the “molar ratio” of IGF1/IGFBP3 that indicates moles of IGF1 present over moles of IGFBP3 available to bind. The molar ratio was calculated considering 1 ng/ml IGF = 0.13 nmol and 1 ng/ml IGFBP3 = 0.036 nmol as reported previously^25^,^47^. Higher IGF1-IGFBP3 molar ratio indicates higher number of IGF1 molecule present in serum compared to molecules of IGFBP3 available to bind, hence indicates unbound IGF1 level^25^,^48^. At twelve months after radiation exposure, although the IGF1 level is similar to control, the IGFBP3 level is lower than the controls (Table 1). Consequently, free IGF1 indicated by increased IGF1/IGFBP3 ratio is higher twelve months after radiation exposure (Table 1). Notably, increasing trend with age of IGF1 levels were observed in 12-month controls relative to 2-month control. Reports in literature on relationship between IGF1 and age are mixed. Although literature suggests decreasing trend of IGF1 with increasing age, there are evidences to the contrary as well. Increased IGF1 in older age has been attributed to altered growth hormone (GH)/IGF1 homeostasis and has the implications for increased risk of carcinogenesis and chronic diseases^25^. Since we observed higher free IGF1 (from IGF1/IGFBP3 ratio) and leptin (described below) levels at 12-month relative to 2-month time point, immunoblot and immunohistochemical assessment of the IGF1/leptin signaling axis were performed in the 12-month post-exposure samples. Expression of IGF1R in intestine (Fig. 2A,B) and colon (Fig. 2C,D) twelve months after radiation exposure showed significantly increased immunostaining compared to controls.

### Increased leptin level was associated with unaltered adiponectin level in serum

Although energetic ^56^Fe radiation did not alter adiponectin levels, we observed significantly increased serum leptin levels two months after exposure (Table 2). As a result of unaltered adiponectin but increased leptin levels, the leptin/adiponectin ratio was increased in two months post-exposure samples (Table 2). Adiponectin levels also remained unchanged twelve months after radiation exposure (Table 2). However, serum leptin level was significantly higher in irradiated samples relative to controls (Table 2). Calculated leptin/adiponectin ratio was significantly higher in 12-month post-irradiated samples (Table 2).

### Energetic ^56^Fe radiation exposure was associated with increased leptin decreased adiponectin receptor expression

**Figure 3:**
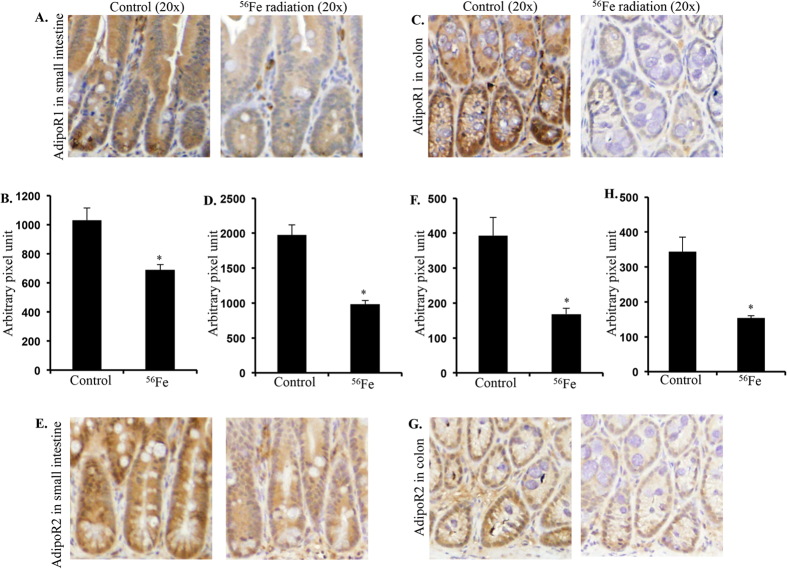
Persistent downregulation of AdipoR1 was observed in intestine (**A,B**) and colon (**C,D**) twelve months after heavy ion radiation exposure. Decreased expression of AdipoR2 was also observed in intestine (**E,F**) and colon (**G,H**) twelve months after radiation exposure.

Expression of Ob-R expression assessed in twelve months post-exposure samples was significantly higher in irradiated intestine (Fig. 2E,F) and colon (Fig. 2G,H) samples relative to controls. In contrast, AdipoR1 expression was lower in both intestine (Fig. 3A,B) and colon (Fig. 3C,D) twelve months after exposure to energetic ^56^Fe ions. Compared to control, decreased expression of AdipoR2 was also observed in intestine (Fig. 3E,F) and colon (Fig. 3G,H) samples from mice exposed to energetic ^56^Fe ions.

### Activation of proliferative pathways downstream of IGF1/leptin signaling axis after energetic ^56^Fe radiation exposure

**Figure 4:**
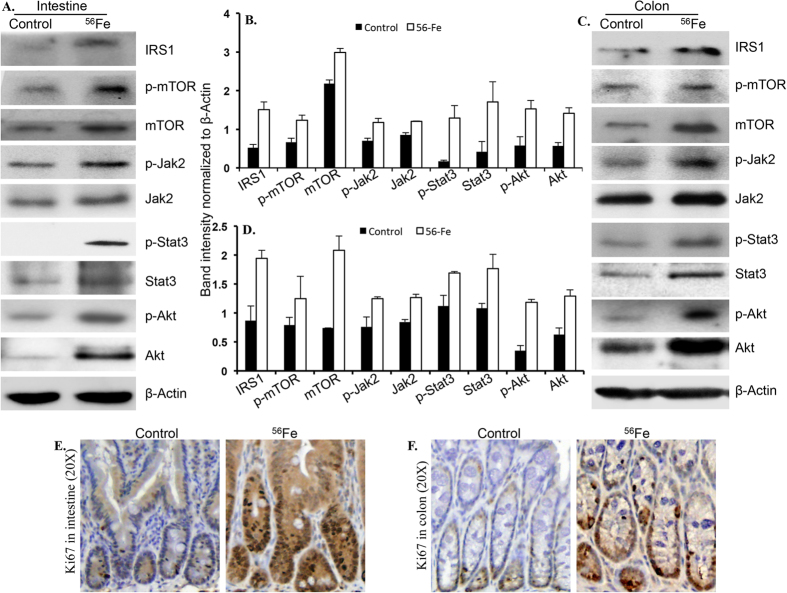
Immunoblot analysis of IRS1, p-mTOR, mTOR, p-Jak2, Jak2, p-Stat3, Stat3, p-Akt, Akt showed activation of IGF1 and leptin signaling in intestine (**A,B**) and colon (**C,D**) 12 months after radiation exposure. Immunostaining of Ki-67 in small intestine sections from irradiated mice showed greater staining relative to control mice (**A,B**). Colon sections from irradiated mice also showed increased Ki67 staining relative to controls (**C,D**). Error bars represent mean ± SEM and p < 0.05 was considered significant, compared to sham irradiated control.

Signaling pathways downstream of IGF1 and leptin were assessed in intestine and colon tissue samples twelve months after exposure to radiation using immunoblot analysis (Fig. 4). Results showed increased expression of IRS1, phospho-mTOR (p-mTOR), mTOR, phospho-Jak2 (p-Jak2), and Jak2 in intestine (Fig. 4A,B) as well as in colon (Fig. 4C,D). Immunoblot analysis also showed increased levels of phospho-Stat3 (p-Stat3), Stat3, phospho-Akt (p-Akt), and Akt in intestine (Fig. 4A,B) and colon samples (Fig. 4C,D). Immunostaining of twelve months post-exposure intestine (Figs 4E and S6A) and colon (Figs 4F and S6B) sections for the proliferative marker Ki67 showed higher staining relative to controls.

## Discussion

Upon observation of increased body weight and obesity-related factors, the current study dissected the leptin/IGF1 signaling axis previously unexplored in relation to heavy ion space radiation in mouse intestine and colon. We have demonstrated increased leptin and IGF1 in serum, and activation of signaling pathways downstream to leptin/IGF1 and consequent increased cell proliferation in tissues up to 12-months after exposure to 1.6 Gy of energetic ^56^Fe radiation. We acknowledge that the dose of ^56^Fe radiation used in the current study is more than the dose astronauts are expected to receive from a long duration space mission. Typically, an astronaut with current shielding and solar quite time (absence of solar particle event or solar storm) is expected to receive one-tenth (0.17 Gy) of the dose used in the current study during a return trip to Mars^49^. However, considering that there is no precedence of heavy ion radiation study involving leptin/IGF1 signaling in mice, we used a higher dose for the initial study that allowed us to dissect this signaling axis known for its role in GI homeostasis and cellular metabolism.

Others and we have reported that radiation exposures profoundly affect hormonal physiology with implications for GI pathologies^20^,^25^,^27^,^28^,^29^,^30^,^31^,^32^,^50^,^51^,^52^. In our previous study, we have reported activation of leptin/IGF1 signaling axis in intestine and colon after exposure to 2 Gy γ radiation. Although the current study also dissected the leptin/IGF1 signaling, it was performed in relation to heavy ion radiation. Here we show for the first time that exposure to energetic ^56^Fe ions activated leptin/IGF1 signaling axis even one year after exposure. While we did observe at least two qualitative/unique differences, our data on γ and heavy ion radiation mostly demonstrated quantitative rather than qualitative differences between the two radiation types. When we compared data from two types of radiation (Figures S1 to S7, Tables S1 and S2), we observed more pronounced molecular responses after heavy ion radiation relative to γ radiation. However, two key factors that distinguished heavy ions from γ-ray responses were increased body weight and decreased intestinal AdipoR1, which were observed only with ^56^Fe radiation. Average body weight and intestinal AdipoR1 in the γ-irradiated group was not altered and therefore, was not presented in the previous publication on γ radiation. In contrast, these two parameters were significantly altered in the heavy ion irradiated group relative not only to controls but also to the γ-irradiated group (Figures S1A and S4A). For serum levels of leptin and IGF1, we observed a mixed response between the radiation types at chosen time points (Tables S1 and S2). Although currently we do not have any data to explain why serum hormone levels were not as distinct as molecular results between the two radiation types, evidences in literature suggest that such mixed leptin/IGF1 results in serum could be due to factors such as insulin and estrous cycle dependent estrogen level, which are known to modulate serum levels of leptin and IGF1^53^,^54^,^55^. Mixed response could also be due to the female mice used in the current study and in the future similar studies in male mice will allow gender comparison for leptin/IGF1 response after heavy ion radiation exposure. Additionally, in view of our earlier study showing remarkable estrogenic response with gamma radiation^56^, it would be worthwhile to further investigate interaction between estrogen and IGF1/leptin after heavy ion radiation to understand this mixed response. Importantly, however, at the signaling level we observed greater activation of leptin/IGF1 signaling after heavy ions relative to γ radiation. Overall, our current data on leptin/IGF1 and previous data on oxidative stress, autophagy, DNA damage response and senescence, and GI tumorigenesis support the notion that heavy ion radiation-induced long-term responses are more pronounced and thus poses more GI risk relative to γ radiation^3^,^4^,^10^,^13^,^14^,^57^.

Leptin and IGF1 are multifunctional hormones which are involved in key GI functions such as motility, nutrient absorption, inflammation, immune and neuroendocrine function, cell survival and apoptosis, and GI epithelial cell proliferation^24^,^58^. Consequently, activation of leptin/IGF1 signaling after ^56^Fe exposure has implications for long-term pathophysiological changes in the GI tract of astronauts. Space flight studies have reported endocrinal alteration including hormones involved in energy metabolism^59^,^60^,^61^,^62^. However, astronauts’ data are mostly from low earth orbit (LEO) space travel (within Van Allen belt) rather than prolonged travel beyond LEO such as planned mission to Mars^62^. Currently, we do not have endocrinal data for prolonged space travel beyond LEO. Additionally, follow up studies in 321 astronauts from 1980 to 2009 recorded seven cancer deaths and one was due to colorectal cancer^63^,^64^. However, considering that astronaut population is small and most data are from LEO missions, drawing statistically robust epidemiological conclusions and estimating risk for future missions beyond LEO are yet to be achieved. In this regard, studies in animal models are expected to provide key biological data necessary to model human risk estimation. Furthermore, along with increased leptin level in serum we also observed increased expression of leptin receptors and activation of Jak2/Stat3 signaling, which is downstream of leptin, in intestine as well as in colon. Importantly, the Jak2/Stat3 pathway is involved in cellular growth and differentiation and is implicated in intestinal homeostasis as well as in colorectal carcinogenesis^65^,^66^,^67^. Specifically, Jak2/Stat3 along with RhoA/ROCK and AMPK pathways, which are downstream to leptin, due to their roles in cellular tight junctions (TJ) could adversely influence intestinal epithelial cell migration as well as intestinal epithelial barrier^66^,^67^. Although we have shown long-term activation of leptin signaling, additional experiments will be required to understand its impact on metabolic functions such as regulation of energy expenditure and glucose metabolism^24^,^66^. Importantly, increased leptin signaling including Jak2/Stat3 has been demonstrated to accompany pathogenesis of CRC^65^ and we have already demonstrated in mouse models increased frequency of intestinal tumorigenesis after heavy ion radiation exposure^4^,^13^. Additionally, leptin true to its multimodal pro-growth effects has also been reported to activate mTOR via Akt^68^ and interestingly, our study demonstrated increased levels of both the phospho-mTOR as well as phospho-Akt after ^56^Fe radiation. Akt is known to activate mTOR activity, which in turn has been reported to upregulate leptin synthesis suggesting a positive feedback for leptin action^23^. In contrast, adiponectin, unlike leptin, is decreased in CRC and reduced adoponectin is considered a CRC risk factor. Indeed, adiponectin opposes leptin function to inhibit cell proliferation and promote apoptosis^21^,^27^,^69^. Unaltered adiponectin level and decreased AdipoR1 and 2 expression levels is expected to allow unopposed leptin action in intestine and colon after heavy ion radiation exposure.

Energetic ^56^Fe radiation exposure also led to increased IGF1 and insulin, and decreased IGFBP3 levels in serum along with increased IGF1R in tissues. Structural similarity between IGF1 and insulin, and IGF1R and insulin receptor (INSR) has been reported and it has also been reported that both the hormones are capable of activating each other’s receptor^53^,^70^,^71^. Although predominantly insulin/INSR affects metabolism and IGF1/IGF1R works on growth and development, both the systems are also known to crosstalk and there is functional overlap^53^,^70^,^71^. Notably, altered expression of IGF1, insulin, IGFBP3, and IGF1R at the mRNA and protein level are independent events and are regulated by a multitude of factors such as estrogen, reactive oxygen species, and platelet derived growth factor (PDGF)^53^,^70^,^71^. These are also regulated by metabolic states and growth and developmental signals. Importantly, 90% of the circulating IGF1 in postnatal serum is bound to IGFBP3 and therefore, free bioavailable IGF1 is mostly determined by IGFBP3, which was the focus of this study. While IGFBP3 was investigated here, additional studies on other IGFBPs could provide a comprehensive understanding of the IGF1/IGFBP/insulin axis in relation to heavy ion radiation exposure. Upon binding to IGF1R, IGF1 initiates a cascade of potent pro-survival and anti-apoptotic signaling pathways including Ras, MAP kinase, and PI3 kinase^25^,^72^. Physiologically, IGF1R plays important roles in growth and development and pathologically, its increased expression has been reported to be involved in diverse disease processes including malignant and immunological diseases^73^. In GI tract, increased IGF1R expression has been reported in gastric^74^ as well as colorectal^75^ carcinogenesis. While most of the its biological effects are due to IGF1 binding, IGF1R can also bind to ligands such as insulin and IGF2 for activation of pro-survival and mitogenic signaling^71^. Interestingly, IGF1 signaling has been reported to cross talk with leptin signaling to enhance cellular proliferation and colorectal carcinogenesis. Reduced IGFBP3 along with increased pro-proliferative Ki67 staining in intestine and colon is in agreement with earlier observations reporting anti-proliferative and pro-apoptotic effects of IGFBP3^76^,^77^. Our observations in this study could act as the prelude necessary for future studies on contribution of leptin/IGF1 signaling axis to some of the heavy ion radiation’s long-term consequences such as premature senescence, metabolic alterations, and GI carcinogenesis we have reported previously^4^,^13^,^57^,^78^,^79^. The current study performed in wild type mice irradiated with a non-lethal dose of ^56^Fe radiation is an initial step towards understanding GI tract related long-term hormonal alterations after heavy ion space radiation exposure especially after low dose exposures.

Apart from carcinogenesis, radiation exposure has been related to obesity in human^41^,^42^,^43^,^44^ as well as in animal^45^ studies. However, this is the first report in mice showing that heavy ion radiation exposure is also associated with obesity, which has been reported as a risk factor for colorectal carcinogenesis. Increased IGF1/leptin signaling, insulin, TG, and PPARγ are suggestive of an altered metabolic state related to lipogenesis, hyperinsulinemia, insulin resistance, and increased lipid accumulation leading to increased body weight after ^56^Fe radiation which has the potential to increase CRC risk after heavy ion radiation. Additionally, IGF1, insulin, leptin, TG, adiponectin, PPARγ, IGF1R, and IGFBP3 investigated in the current study have all been implicated in colorectal carcinogenesis and have been proposed as CRC risk markers^21^,^25^,^27^,^28^,^29^,^47^,^80^,^81^,^82^,^83^. However, related to this first report is the broader question of whether the obesity-associated CRC risk factors could also be used as markers for heavy ion radiation-related CRC development and this would require additional studies.

## Materials and Methods

### Mice and irradiation

Mice (6 to 8 wks, female, C57BL/6J) were purchased from Jackson Laboratories (Bar Harbor, ME) and delivered directly to Brookhaven National Laboratory (BNL) animal facility. Mice (n = 9 mice/group) were exposed to 1.6 Gy ^56^Fe radiation (whole body single exposure; energy: 1000 MeV/n; LET: 148 keV/μm) at the NASA Space Radiation Laboratory (NSRL) located in BNL. The dose of 1.6 Gy was chosen from our previous survival experiments in this mouse strain and was equitoxic to 2 Gy of γ radiation^3^. For ^56^Fe irradiation, mice were placed in small transparent rectangular Lucite boxes (7.6 cm × 3.8 cm × 3.8 cm) with multiple holes for air circulation and post-irradiation mice were returned to their home cages and housed in an air-conditioned room with 12-hour dark and light cycle maintained at 22 °C in 50% humidity and monitored daily. Heavy ion radiation dosimetry was determined by the NSRL physics team and mice were exposed to constant LET by placing them at the entrance plateau region of the Bragg curve^84^,^85^,^86^,^87^. Mice were shipped from BNL to Georgetown University (GU) animal facility on the day after irradiation early in the morning in a temperature-controlled environment along with the respective sham irradiated control groups for same day delivery. Animal facilities at BNL and GU are AAALACI (Association for Assessment and Accreditation of Laboratory and Animal Care International) accredited facilities. All animal procedures including irradiation were performed according to protocols approved by the Georgetown University Animal Care and Use Committees (Approved protocol# 16-006-100257) and BNL Animal Care and Use Committee (Approved protocol#345). All animals were provided certified rodent diet with filtered water *ad libitum*. Any mouse with declining health determined by using the parameters such as hunched posture, ruffled fur, diarrhea, reduced activity, and weight loss (>15%) was euthanized by CO_2_ asphyxiation and was excluded from the specific study group. Our research followed Guide for the Care and Use of Laboratory Animals, prepared by the Institute of Laboratory Animal Resources, National Research Council, and U.S. National Academy of Sciences.

### Serum and tissues

Mice were euthanized two or twelve months after radiation and serum samples were obtained by immediate cardiac puncture of the euthanized animal using sterile 1 ml syringes and 25G needles. Since we were interested in understanding long-term effects of radiation on gastrointestinal tissues, we chose a relatively early time point (2-month) and a late time point (12-month) for our study. Additionally, the two time points were chosen because the first time point represents early adult life (3 to 6 months) and the second time point represents mid adult life (10 to 14 months)^88^. The goal was to see if radiation-induced biological alterations that are observed in younger adult are detected later in middle age for persistence of effects. Mice were weighed before euthanizing at twelve months post-exposure time-point. Serum samples were flash frozen in liquid nitrogen and stored at −80 °C for further use. Intestine and colon tissue were surgically dissected out and lumen washed with phosphate-buffered saline (pH = 7.4). Tissues from small intestine (jejunal area) and colon were either fixed in 10% buffered formalin for immunohistochemistry or flash frozen in liquid nitrogen and stored at −80 °C for immunoblots and PCR.

### Serum insulin-like growth factor-1 (IGF-1) estimation

Serum IGF1 concentrations in age matched control and irradiated mice were measured using mouse IGF1 Quantikine ELISA kit (Cat# MG100, R&D systems, Minneapolis, MN) as per manufacturer’s instruction. The assay has a detection sensitivity of 8.4 pg/ml.

### Serum Insulin-like growth factor binding protein 3 (IGFBP3) estimation

Serum IGFBP3 concentrations in age matched control and irradiated mice were measured using mouse IGFBP3 ELISA kit (Cat# Ab100692, Abcam, Cambridge, MA) as per manufacturer’s instruction. The assay has a detection sensitivity of <70 pg/ml.

### Serum insulin estimation

Serum total insulin level was measured using Mouse Insulin ELISA kit (Sigma-Aldrich, Saint Louis, MO, USA) as per manufactures’ instructions. In brief, serum samples were diluted 1:4 in sample dilution buffer and 100 μl of diluted samples and standard were placed in a pre-coated ELISA plate for 2.5 h at room temperature with gentle shaking. Following multiple washes detection was done using biotin-streptavidin-HRP (horse-radish peroxidase) and TMB (tetramethylbenzidine) reagent and absorbance was recorded at 450 nm. Serum insulin levels were estimated using a standard curve (range 6.25 μIU/ml to 400 μIU/ml). Both samples and standard were used in duplicates for intra assay variation.

### Serum triglyceride (TG) estimation

Serum triglyceride was measured using Triglyceride Colorimetric assay kit (Cayman chemical company, Ann Arbor, MI, USA) as per manufacture’s instructions. In brief, 10 μl of mouse serum was placed in each well of 96 well plate and reaction was initiated using enzyme buffer solution and incubated at room temperature for 15 min and absorbance was recorded at 535 nm. Serum TG levels were estimated using a standard curve (range 3.125 mg/dl to 200 mg/dl). Both samples and standard were used in duplicates for intra assay variation.

### Serum leptin estimation

Serum leptin concentrations in age matched control and irradiated mice were measured using mouse leptin ELISA kit (Cat# KMC2281, Life Technologies, Grand Island, NY) as per manufacturer’s instruction. The assay has a detection sensitivity of <50 pg/ml.

### Serum adiponectin estimation

Serum adiponectin concentrations in age matched control and irradiated mice were measured by using mouse adiponectin ELISA kit (Cat# KMP0041, Life Technologies, Grand Island, NY) as per manufacturer’s instruction. The assay has a detection sensitivity of <50 pg/ml.

### Real time quantitative PCR

Total RNA (1 μg) isolated from intestinal and colonic tissues was used for cDNA preparation using the iScript cDNA synthesis kit (Bio-Rad, Hercules, CA, USA). The mRNA expression was analyzed using real-time polymerase chain reaction containing SsoAdvanced™ universal SYBR green supermix (Bio-Rad), 5 ng cDNA, and 0.2 μM primers (PPARγ-forward primer 5′GATAAAGCATCAGGCTTCCA3′ and reverse primer 5′TGATGGCATTGTGAGACATC3′) in 20 μl reaction volume. Reactions were setup on CFX96 real-time system (Bio-Rad) with thermal settings: 95 °C for 5 min and then 45 cycles of 95 °C for 15 s, annealing/extension at 58 °C for 1 min. Relative fold change in gene expression was calculated using β-actin (forward primer-5′AGGTCATCACTATTGGCAAGGA3′ and reverse primer-5′CACTTCATGATGGAATTGAATGTAGTT3′) as endogenous control following the comparative Ct method.

### Immunoblot analysis

Frozen small intestine and colon tissue samples were lysed in ice-cold lysis buffer (0.5% sodium deoxycholate; 0.5% NP-40; 10 mM EDTA in PBS) containing protease inhibitor cocktail (Sigma-Aldrich, St. Louis, MO). Lysates were centrifuged at 12000 × g at 4 °C for 15 min and immunoblots were performed using appropriate primary antibodies (IRS1: dilution-1:500, 611394, BD Biosciences, San Jose, CA; mTOR: dilution-1:1000, PA5-17780, Thermo Scientific, Pittsburgh, PA; phospho-mTOR: dilution-1:500, 2971s, Cell Signaling Technology, Danvers, MA; JAK2: dilution-1:200, Sc7229, Santa Cruz Biotechnology; phospho-JAK2: dilution-1:200, Sc21870, Santa Cruz Biotechnology; phospho-STAT3: dilution-1:400, Sc8059, Santa Cruz Biotechnology, Dallas, TX; STAT3: dilution-1:500, Sc482, Santa Cruz Biotechnology, Dallas, TX; AKT: dilution-1:500, Sc5298, Santa Cruz Biotechnology, Dallas, TX; phospho-AKT: dilution-1:500, 9277S, Cell Signaling Technology, Danvers, MA; β-Actin: dilution-1:2500, Sc47778, Santa Cruz Biotechnology). Horseradish peroxidase (HRP) conjugated secondary antibody and enhanced chemiluminescence (ECL) detection system (Thermo Fisher Scientific, Rockville, MD) were used to develop immunoblots. Images captured on x-ray films were scanned and used for densitometric quantification by ImageJ v1.46 software (National Institutes of Health, Bethesda, MD). Band intensity was normalized to β-actin band intensity in respective column and data from nine mice are expressed as mean ± standard error of mean (SEM) and a representative image for each protein in presented in the results.

### Immunohistochemistry

For immunohistochemistry, 4 μm thick sections were prepared after paraffin embedding of fixed tissues. Immunohistochemistry was performed for Ki67 (Catalog# Sc15402; dilution 1:50; Santa Cruz Biotechnology, Santa Cruz, CA), IGF1R (Catalog# sc-7952; dilution 1:50; Santa Cruz Biotechnology), AdipoR1 (Catalog# sc-99183; dilution 1:50; Santa Cruz Biotechnology) and AdipoR2 (Catalog# sc-99184; dilution 1:50; Santa Cruz Biotechnology), and leptin receptor (Catalog# sc-1834; dilution 1:40; Santa Cruz Biotechnology) following antigen retrieval using citrate buffer (pH 6.0). SuperPicture 3rd -Generation IHC detection kit (Catalog No. 87-9673; Invitrogen, Carlsbad, CA) was used for signal detection and color development. Five slides from five mice in each group were stained for each protein and a representative image from one animal is presented in results. To determine the specificity of the staining, appropriate controls were run in parallel with the experimental slides.

### Image acquisition and statistical analysis

Immunohistochemistry images were captured using bright field microscopy at 20x microscopic magnification and twenty randomly selected visual fields were captured in each study group for quantification. Quantification was performed using ImageJ v1.46 software as per protocol described earlier^10^,^89^,^90^. In addition, Image-based Tool for Counting Nuclei (ITCN) plug-in for ImageJ was used for counting Ki67 positive nuclei. Statistical significance between two groups were determined using two-tailed paired student’s t-test and the level of significance was set at p < 0.05. Error bar represent mean ± standard errors of the mean (SEM).

## Additional Information

**How to cite this article**: Suman, S. *et al*. Space radiation exposure persistently increased leptin and IGF1 in serum and activated leptin-IGF1 signaling axis in mouse intestine. *Sci. Rep.* **6**, 31853; doi: 10.1038/srep31853 (2016).

## Supplementary Material

Supplementary Tables and Figures

## Figures and Tables

**Table 1:** Serum levels of IGF1 and IGFBP3 (±SEM).

	IGF1 (ng/ml)		IGFBP3 (ng/ml)		IGF1/IGFBP3 ratio	
	2 m	12 m	2 m	12 m	2 m	12 m
Control	402.4 ± 14.4	559.4 ± 23.3	1940.4 ± 48.3	2503.2 ± 38.9	0.75 ± 0.03	0.81 ± 0.04
^56^Fe ions	486 ± 8.1*	579.8 ± 51.3	2170.9 ± 45.7	1912.4 ± 44.7*	0.81 ± 0.02*	1.14 ± 0.05*

^*^Indicates significant (p < 0.05) difference relative to control.

**Table 2:** Serum levels of leptin and adiponectin (±SEM).

	Leptin (ng/ml)		Adiponectin (μg/ml)		Leptin/Adiponectin ratio	
	2 m	12 m	2 m	12 m	2 m	12 m
Control	7.2 ± 0.8	20.6 ± 3.6	177.2 ± 10.8	218.1 ± 9.0	0.04 ± 0.003	0.09 ± 0.01
^56^Fe ions	16.2 ± 3.8*	34.5 ± 7.1*	180.3 ± 9.4	198.4 ± 13.6	0.08 ± 0.01*	0.19 ± 0.03*

^*^Indicates significant (p < 0.05) difference relative to control.
