# Supplementary material for: Space radiation exposure persistently increased leptin and IGF1 in serum and activated leptin-IGF1 signaling axis in mouse intestine
Source: Sci Rep. 2016 Aug 25;6:31853. doi: 10.1038/srep31853 (PMC4997262; doi:10.1038/srep31853)
Supplement: Supplementary Tables and Figures [file srep31853-s1.pdf]

Space radiation exposure persistently increased leptin and IGF1 in serum and activated leptin-IGF1 signaling axis in mouse intestine

Shubhankar Suman[1], Santosh Kumar[1], Albert J Fornace Jr.[1], Kamal Datta[1*]
[1]Department of Biochemistry and Molecular & Cellular Biology and Lombardi Comprehensive Cancer Center, Georgetown University, Washington, DC 20057, USA.

Supplementary methods for γ-irradiation. Mice (C57BL/6J, female, 6-8 week old; Jackson Laboratories, Bar Harbor, ME, USA) were housed at the Georgetown University' s (GU) animal facility and animal procedures were approved by GU Animal Care and Use Committee (GUACUC). Foe γ-irradiation, mice were placed in a circular pie shaped, well-ventilated plastic mouse holder. The mouse holder with mice was placed on a rotating turntable inside the irradiator, and mice were exposed to γ radiation using a ^{137}Cs source and a total dose of 2 Gy was delivered to the whole body. Control groups were sham irradiated and after irradiation mice were returned to their home cage, and monitored regularly.

Supplementary Table 1. Serum levels of IGF1 and IGFBP3 (±SEM)

	IGF1 (ng/ml)		IGFBP3 (ng/ml)		IGF1/IGFBP3 ratio	
	2 m	12 m	2 m	12 m	2 m	12 m
Control	402.4±14.4	559.4±23.3	1940.4±48.3	2503.2±38.9	0.75±0.03	0.81±0.04
γ-rays	*449.2±9.7	*600.8±23.8	*1776.1±63.1	*1864.1±80.1	*0.91±0.01	*1.16±0.01
^{56}Fe ions	*486±8.1	579.8±51.3	2170.9±45.7	*1912.4±44.7	*0.81±0.02	*1.14±0.05

*Indicates significant (p<0.05) difference relative to control.

Supplementary Table 2. Serum levels of leptin and adiponectin (±SEM)

	Leptin (ng/ml)		Adiponectin (μg/ml)		Leptin/Adiponectin ratio	
	2 m	12 m	2 m	12 m	2 m	12 m
Control	7.2±0.8	20.6±3.6	177.2±10.8	218.1±9.0	0.04±0.003	0.09±0.01
γ-rays	*14.1±2.2	*54.4±14.2	198.5±8.6	208.0±19.8	*0.06±0.008	*0.16±0.21
^{56}Fe ions	*16.2±3.8	*34.5±7.1	180.3±9.4	198.4±13.6	*0.08±0.01	*0.19±0.03

*Indicates significant (p<0.05) difference relative to control.

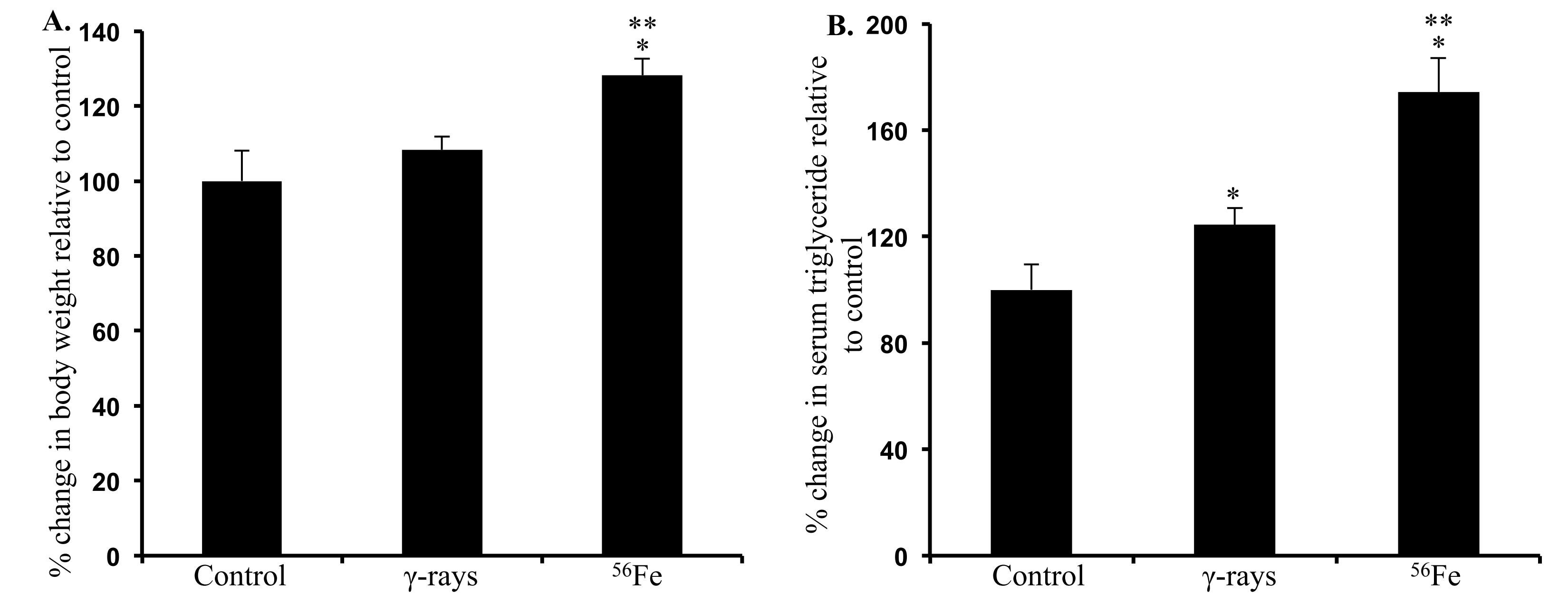

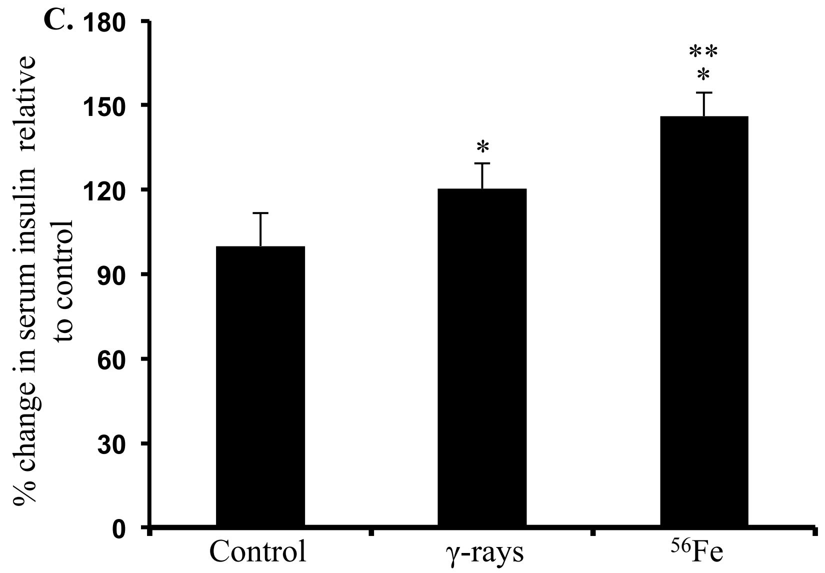

Supplementary Figure S1. Increased adiposity 12-month after exposure to ^{56}Fe radiation. A) Higher body weight in ^{56}Fe-irradiated mice relative to control and γ-irradiated mice. However, body weight in γ-irradiated group was not significantly different relative to control. B) Increased serum triglyceride (TG) after ^{56}Fe radiation. C) Increased serum insulin after ^{56}Fe radiation. *$p<0.05$ relative to control, **$p<0.05$ relative to γ radiation.

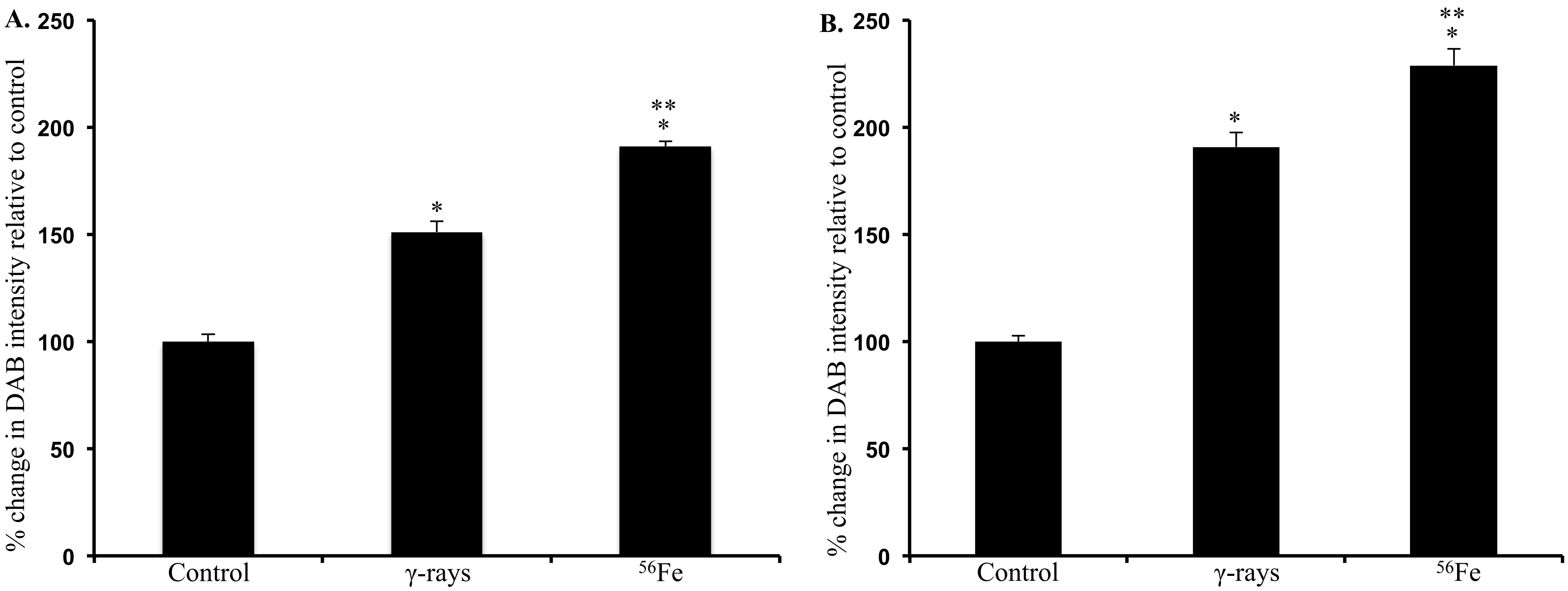

Supplementary Figure S2. Comparison (% change in irradiated groups taking control as 100%) of IGF1R staining quantification in intestinal (A) and colon (B) sections 12-month after exposure to γ and ^{56}Fe radiation. Greater increase in IGF1R was observed after ^{56}Fe relative to control and γ radiation. *$p<0.05$ relative to control, **$p<0.05$ relative to γ radiation.

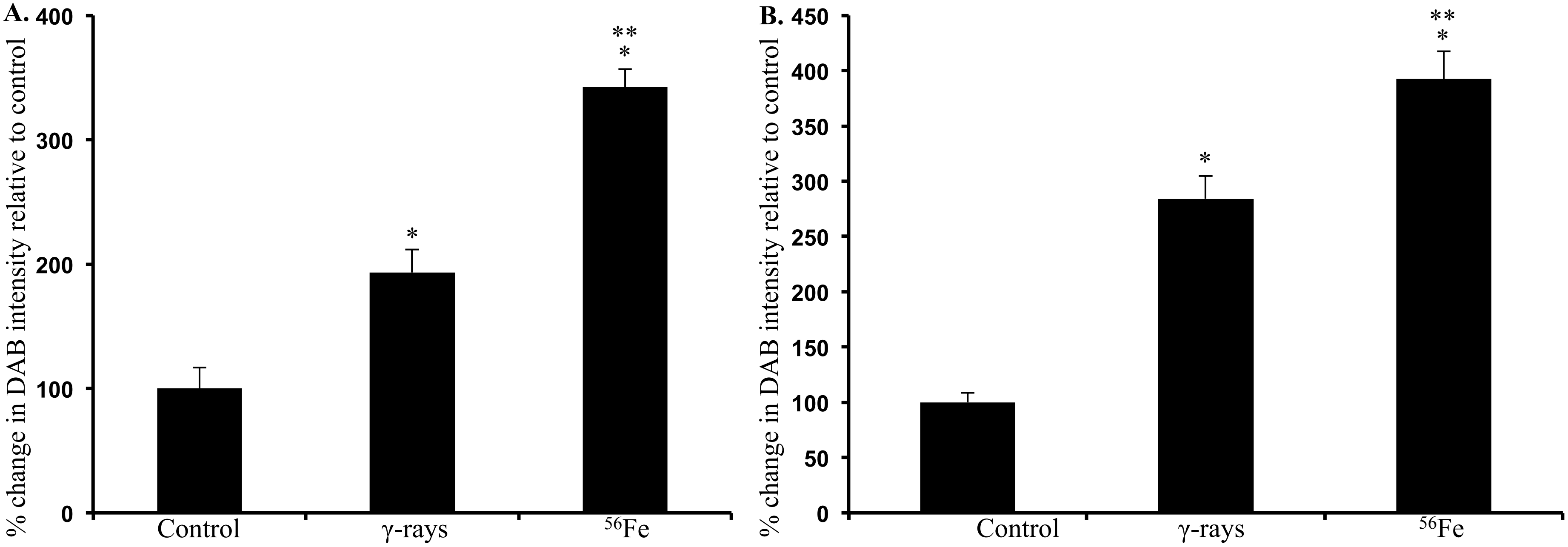

Supplementary Figure S3. Comparison (% change in irradiated groups taking control as 100%) of Ob-R staining quantification in intestinal (A) and colon (B) sections 12-month after exposure to γ and ^{56}Fe radiation. Greater increase in Ob-R was observed after ^{56}Fe relative to control and γ radiation. *p<0.05 relative to control, **p<0.05 relative to γ radiation.

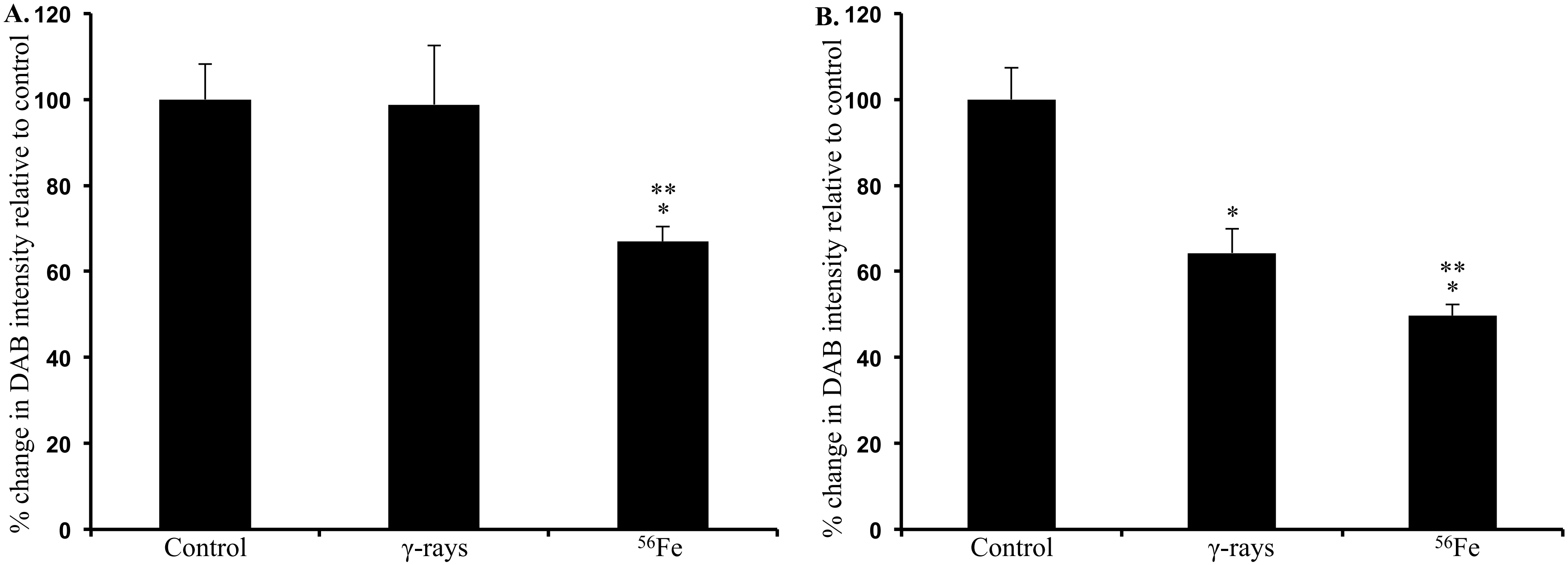

Supplementary Figure S4. Comparison (% change in irradiated groups taking control as 100%) of AdipoR1 staining quantification in intestinal (A) and colon (B) sections 12-month after exposure to γ and ^{56}Fe radiation. Greater increase in AdipoR1 was observed after ^{56}Fe relative to control and γ radiation. However, γ radiation did not significantly alter AdipoR1 staining in intestine relative to control. *$p<0.05$ relative to control, **$p<0.05$ relative to γ radiation.

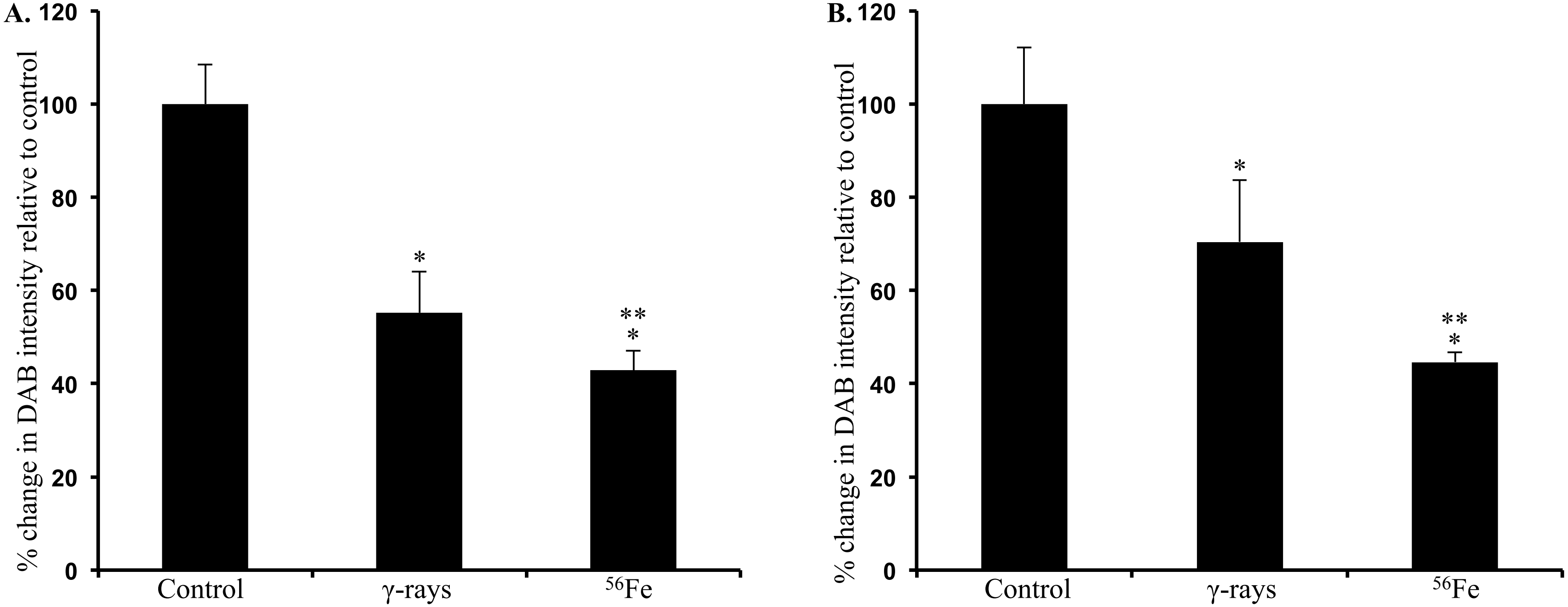

Supplementary Figure S5. Comparison (% change in irradiated groups taking control as 100%) of AdipoR2 staining quantification in intestinal (A) and colon (B) sections 12-month after exposure to γ and ^{56}Fe radiation. Greater increase in AdipoR2 was observed after ^{56}Fe relative to control and γ radiation. *$p<0.05$ relative to control, **$p<0.05$ relative to γ radiation.

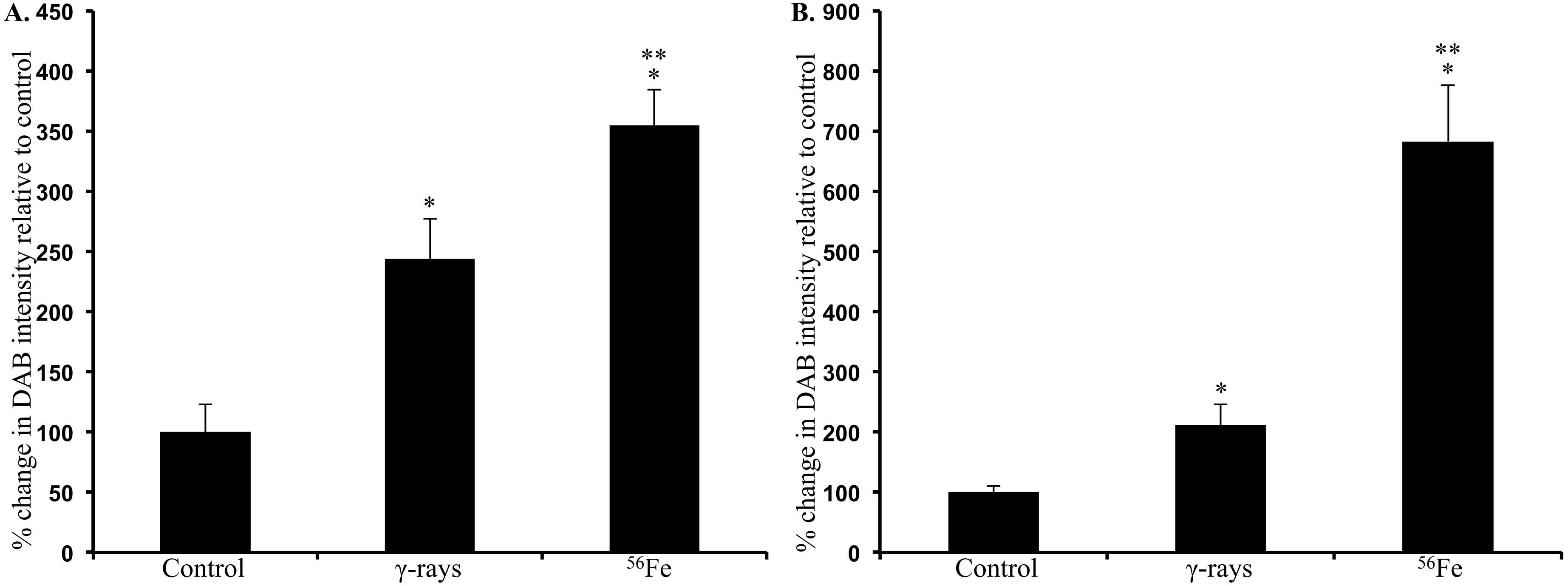

Supplementary Figure S6. Comparison (% change in irradiated groups taking control as 100%) of Ki67 staining quantification in intestinal (A) and colon (B) sections 12-month after exposure to γ and ^{56}Fe radiation. Greater increase in Ki67 was observed after ^{56}Fe relative to control and γ radiation. *p<0.05 relative to control, **p<0.05 relative to γ radiation.

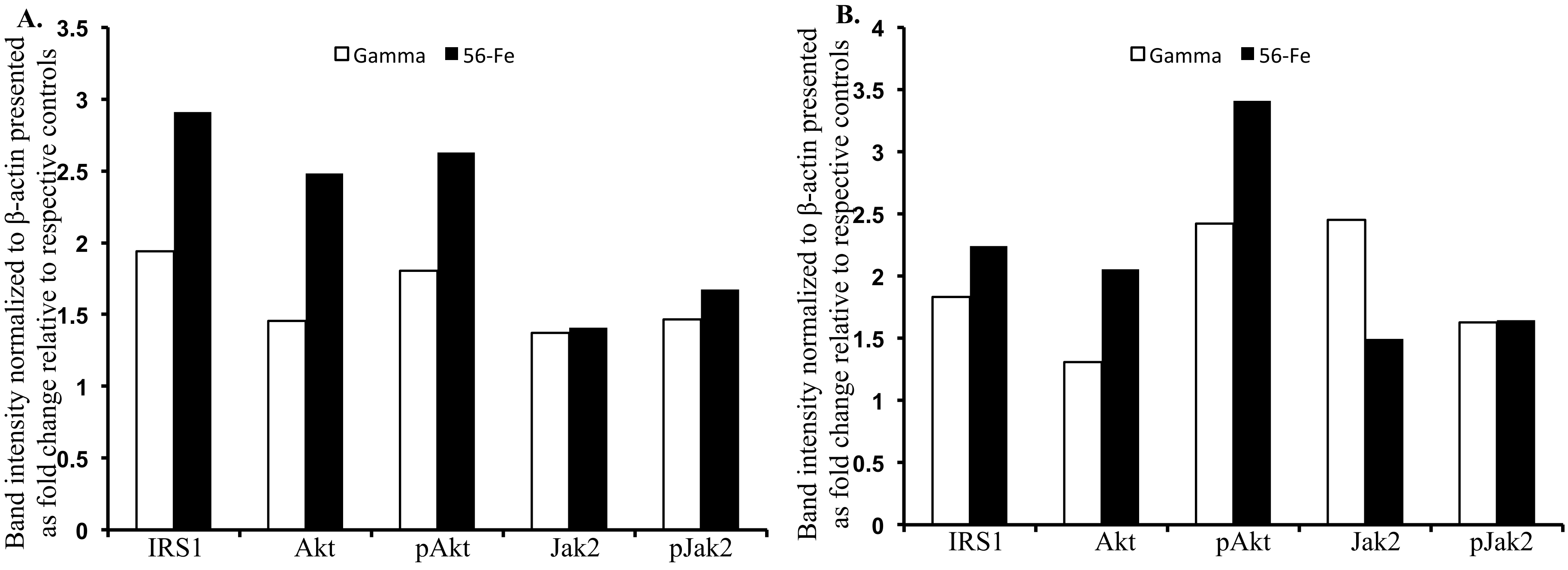

Supplementary Figure S7. Comparison of immunoblot fold change quantification in intestinal (A) and colon (B) sections after exposure to γ and ^{56}Fe radiation. Overall, greater alterations in key signaling molecules were observed after ^{56}Fe relative to γ radiation.
